# Supplementary material for: A longitudinal study of perceived stress and cortisol responses in an undergraduate student population from India
Source: PLoS One. 2021 Jun 4;16(6):e0252579. doi: 10.1371/journal.pone.0252579 (PMC8177861; doi:10.1371/journal.pone.0252579)
Supplement: Attachment — Submitted filename: Response to Reviewers comments final.docx [file pone.0252579.s003.docx]

**Dear Editor and Reviewers,**

Please find responses to your comments below:

**Responses to Editor’s comments:**

**Throughout the article the term "sex difference" is used. I suggest to use gender-difference, since the authors themselves point to non-biological factors potentially causing the increase in cortisol in women. Or gender-/sex-difference if they which to point to potential biological differences in the stress-response by sex.**

Point well taken, in the revised manuscript gender/sex difference has been used.

**Impact of "stressors" were assessed in stratified analyses over all time points. As far as I understand they did not address interactions of sex and stressors.**

We did look at sex and stressors interaction, but it was not significant. Please see lines 217-226:

There were no sex differences in the types of stressors reported (z=-0.66, p=0.509), but different type of stressors significantly differed across time. We thus divided the data across sexes and performed separate mixed effect models to understand how the stressors were different across time points.

Total number of stressors reported by both men and women were lowest at time point 4 compared to time point 1 (men: z=-2.5, p=0.012, Fig. 2a; women: z=-2.86, p=0.004, Fig. 2b). Additionally, men also reported a significantly lower number of stressors at time point 5 compared to 1 (z=-2.23, p=0.025, Fig. 2a).

**Please address the limitations in more detail: sample size, missing confounders; as well as the validity of cortisol measures. The authors should also discuss the generalizability of the results and how unmeasured confounders might impact the results, especially from a gender point of view.**

These points have been addressed in the concluding paragraph (lines 314-326) of the revised manuscript.

**Responses to the Reviewers’ comments:**

**Reviewer 1:**

**A. Participants (page 5, lines 91-101):**

**A1. The authors should provide some further information on the selection process, i.e. sampling procedure. How were students recruited?s**

An initial meeting with participants after their first few weeks of joining the semester was done where the researcher explained the course of the study and a research assistant obtained informed consent. The participants completed demographic and psychological questionnaires and were then instructed about the standardized collection of saliva samples according to the study protocol. Participants were asked not to brush their teeth or eat at least 30 minutes before sampling (lines 98-103)

**A2. The authors state that 25 students participated in the study and six measurements were available for ~20 individuals. Please indicate whether patients were included and analyzed on an intention-to-treat basis and if so how many observations were present at each time point. If only individuals with complete measurements were included, please justify. What do the authors mean by ~ (approximately) 20 individuals? Please specify precisely for how many subjects measurements were available at all time points.**

Sample sizes across time points: We had different sample sizes across time points and across physiological and psychological measures.

Time 1: Cortisol data: N=23 (F=16, M=7); Psychological data: N=18 (F=11, M=7)

Time 2: Cortisol data: N=24 (F=16, M=8); Psychological data: N=25 (F=16, M=9)

Time 3: Cortisol data: N=21 (F=16, M=5); Psychological data: N=23 (F=15, M=8)

Time 4: Cortisol data: N=25 (F=16, M=9); Psychological data: N=24 (F=17, M=7)

Time 5: Cortisol data: N=24 (F=17, M=7); Psychological data: N=24 (F=17, M=7)

Time 6: Cortisol data: N=23 (F=16, M=7); Psychological data: N=23 (F=16, M=7)

This information has been added in the text (lines – 124-129 )

**A3. Please justify your sample size.**

To the best of our knowledge, this is the first study of its kind in the Indian population and was a preliminary investigation looking at the correlation between physiological and psychological correlates of stress. We started with 34 individuals. However, since the batch had a low number of male students, we could not increase our male sample size. The initial number of males was 9, and even at a later time, 8-9 males continued to participate in the study, indicating that most females dropped out. Though the sample size is small, the total salivary samples were 140. It was also quite challenging to follow up with students for 6 points of time (1 year). The cost and time associated with taking a larger sample, buying salivary cortisol kits, and analysing them in the lab were other factors that determined our sample size. These are some limitations of our current study (which we have now added in detail), but we hope to address these in future studies.

**B. Discussion:**

**B1. (page 13, line 285): There is a typo in the suicide incidence rate, i.e. “1,00,000” should probably read “100,000”.**

Thanks for pointing this out, we have made the change in the revised manuscript

**Reviewer 2:**

**First and foremost, a major limitation is the small sample size: n = 25 total with nMen = 7, nWomen = 15 across the study period. The uneven and small sample size for men (n= 7) is particularly of concern given that the main results focus on sex differences.**

This concern has been addressed in the response given to the first reviewer justifying the small sample size. Please see lines (51-61) above.

**My second concern is the measurement of cortisol, which was obtained at 0800-0830 h. Without more detailed information on wake time and sleep, it is unclear what a cortisol value at this specific time means. Differences in wake time need to be taken into account with this data.**

We had no control over the sleep cycle and whether students experienced a sound sleep or the exact duration of sleep either. Wake time of most students were within an hour before the sample collection (we asked wake time for some students during sample collection). We understand the data limitation here as we would not know where in the cortisol daily cycle we are actually sampling. However, we think that the repeated sampling design might take care of this limitation to a large extent.

**The authors review evidence for the increased risk for suicide in Asian young adults but did not include information regarding depressive symptoms or suicidal ideation over the study period. If available, such data could strengthen the paper.**

In the current sample we did not have any students who showed depressive symptoms or suicidal ideation over the study period. (lines- 188-189) in the revised manuscript.

**How did the authors ensure that no consumption of food/drink or teeth brushing was conducted half an hour before providing the salivary sample?**

We instructed students specifically on this criterion and confirmed it before sample collection. This has been added in the methods ( lines 98-103) in the revised manuscript.

**The authors name “self-imposed” stress as a reason for higher overall and maintained stress levels, but also mention high parental pressure. I would suggest rephrasing this word, since self-imposed implies that there is no external/social pressure. I might also add that social/cultural expectations to succeed might be particularly prominent for young women.**

This is a relevant point; the word has been rephrased and a relevant research study has been added in the revised manuscript. (please see lines 263-266)
