# Supplementary material for: A longitudinal study of perceived stress and cortisol responses in an undergraduate student population from India
Source: PLoS One. 2021 Jun 4;16(6):e0252579. doi: 10.1371/journal.pone.0252579 (PMC8177861; doi:10.1371/journal.pone.0252579)
Supplement: Attachment — Submitted filename: Response to Reviewers 12521.docx [file pone.0252579.s004.docx]

**Dear Editor and Reviewers,**

Please find responses to your comments below:

**Responses to Editor’s comments:**

**Editor:** Thank you for addressing the reviewer's points and requests for improvement.
One last point, should still be considered, you answered the reviewer 2 point on cortisol measurement (see below), however, you did not take it up in the discussion. It seems a limitation worth mentioning, especially as you did ask for wake time (add to methods):
"We had no control over the sleep cycle and whether students experienced a sound sleep or the exact duration of sleep either. Wake time of most students were within an hour before the sample collection (we asked wake time for some students during sample collection). We understand the data limitation here as we would not know where in the cortisol daily cycle we are actually sampling. However, we think that the repeated sampling design might take care of this limitation to a large extent. "

Please introduce this limitation and your arguments into the manuscript.

**Response:** a line [ lines 103- 104] on wake time has been added to the method section in the main manuscript.

Data limitation about cortisol measurement not knowing the wake time has been included in the discussion section as suggested [lines 318-324] in the manuscript.
